# Meta‐analysis of peripheral mean platelet volume in patients with mental disorders: Comparisons in depression, anxiety, bipolar disorder, and schizophrenia

**DOI:** 10.1002/brb3.3240

**Published:** 2023-08-29

**Authors:** Zhichao Chen, Jing Wang, Ciriaco Carru, Stefania Sedda, Alessandra Matilde Nivoli, Zhi Li

**Affiliations:** ^1^ Department of Biomedical Sciences University of Sassari Sassari Italy; ^2^ Department of Cardiology Second Affiliated Hospital of Shantou University Medical College Shantou China; ^3^ Department of Obstetrics and Gynecology Second Affiliated Hospital of Shantou University Medical College Shantou China; ^4^ Department of Medical, Surgical and Experimental Sciences University of Sassari Sassari Sardegna Italy; ^5^ Psychiatric Unit Clinic of the University Hospital Sassari Sardegna Italy; ^6^ Department of Cardiology The First Affiliated Hospital of Shantou University Medical College Shantou China

**Keywords:** inflammation, mean platelet volume, mental disorders, mental illness

## Abstract

**Background:**

There is a growing interest in the role of immune and inflammatory responses in mental disorders (MDs). Mean platelet volume (MPV) is an extensively utilized hemogram parameter that reflects systemic inflammation and immune function. Our research sought to determine whether a connection exists between MPV and various types of MDs.

**Methods:**

We searched PubMed, EMBASE, PsychINFO, and Web of Science for eligible studies from inception to 15 February 2023, supplemented by manual searching the references from relevant articles. We applied standardized mean difference (SMD) and its 95% confidence interval (CI) to estimate the differences in MPV values in patients with MDs compared to controls.

**Results:**

We analyzed data from 24 surveys with 4843 participants (2450 patients with MDs and 2393 healthy controls). Two‐step meta‐analyses were conducted to estimate the SMD in MPV value between individuals with and without MDs. Higher MPV values were substantially linked to MDs (i.e., depression, anxiety, bipolar disorder, and schizophrenia). Moderator and stratified analyses revealed that the aggregate effects were more robust in specific populations, such as younger patients and those who had not taken antipsychotic medication within the previous month.

**Conclusions:**

Our findings corroborate the role of inflammatory response in the pathogenesis of MDs and the pharmacological treatment of these conditions. Regarding the considerable heterogeneity among studies, the level of evidence was very low to moderate.

## Graphical abstract

1. An inflammatory response is involved in the pathogenesis of mental disorders.
2. Mean platelet volume is a valid indicator of the body‘s inflammatory response in patients with mental disorders.
3. Mean platelet volume level declines during antipsychotic treatment. Age negatively modulates the association between mean platelet volume and mental disorders.

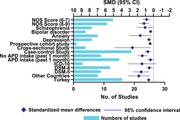

## 1 INTRODUCTION

*Mental disorders* (MDs) are syndromes characterized by considerable impairments in a person's emotional regulation, cognition, or behavior that reflect severe disabilities of psychological, biological, or developmental processes (World Health Organization, 2022). MDs are one of the leading causes of global disease burden, which affect approximately 29% of individuals worldwide during their lifetimes (Steel et al., 2014; Vos et al., 2020). Meanwhile, it was reported that 14.3% of deaths globally, or specifically 8 million deaths per year, were related to MDs (Walker et al., 2015). More recently, increasing studies have reported the mortality rate of common MDs with specific diagnoses (e.g., depression, anxiety, bipolar disorder [BD], and schizophrenia [SCZ]) (Crump et al., 2013; Gilman et al., 2017; Olfson et al., 2015; Pratt et al., 2016).

Depression, anxiety, BD, and SCZ are the most prevalent MDs, with lifetime prevalence rates of 4%, 33.7%, 2.4%, and 1.1%, respectively (Bandelow & Michaelis, 2015; GBD 2015 Disease and Injury Incidence and Prevalence Collaborators, 2016; Grande et al., 2016; Simeone et al., 2015). A recent meta‐analysis of large sample sizes with 2798 healthy controls and 3212 major depressive disorder (MDD) patients reveals the increased level of tumor necrosis factor (TNF), interleukin 6 (IL‐6), and IL‐10 in MDD patients, whereas interferon‐γ is reduced compared to healthy controls (Köhler et al., 2017). The application of spinal cord clip compression to rats was found to induce anxiety and depression‐like behaviors by a significant increase in the level of pro‐inflammatory cytokines (IL‐6 and TNF) in the plasma (do Espírito Santo et al., 2019). Patients with BD have been verified to have an expansion of Toll‐like receptors in peripheral lymphocytes and monocytes (Wieck et al., 2016). Furthermore, a higher level of NLR family pyrin domain containing 3 (NLRP3) was found in the frontal cortex of BD subjects and associated with elevated levels of TNF, IL‐6, IL‐1, and IL‐10 (Kim et al., 2016). Recently, cohort and meta‐analysis studies indicated increased serum concentrations of Cu, K, P, Al and lower Zn, and Se concentrations were detected in untreated BD and SCZ patients compared to healthy controls (Saghazadeh et al., 2020; Santa et al., 2020). It was well established that an imbalance of trace elements may lead to an increase in oxidative stress and impaired immune function. Thus, the pathogenesis of MDs has been conclusively associated with elevated inflammatory responses.

The mean platelet volume (MPV) is one of the parameters that reflects the size and reactivity of circulating platelets. MPV is a low‐cost biomarker and is automatically calculated from the complete blood count in clinics and laboratories. MPV has been utilized as a widely accessible marker of thrombosis and systemic inflammation in numerous studies, including those involving cardiovascular diseases (Sansanayudh et al., 2014), autoimmune diseases (Yavuz et al., 2014), and infections (Zareifar et al., 2014). Recent studies have investigated the association between MPV values and MDs (Almis & Aksoy, 2018; Canan et al., 2012). However, the findings of the relationship between MVP values and MDs were not inconsistent. Apparently, due to inconsistencies in the population, errors between measurement instruments, and differences in statistical power, the variability in results across studies is inevitable.

In this study, we aimed to summarize current evidence that systematically reported the association between MPV and MDs and provide comprehensive findings of MPV values related to MDs. In addition, we intended to determine whether specific MD diagnoses, sex, age, regions, diagnostic instruments, and prior antipsychotic drug (APD) use influenced the association.

## 2 MATERIALS AND METHODS

Our review protocol was registered at the International Prospective Register of Systematic Reviews (ID: CRD42023401244). We reported all procedures following the Preferred Reporting Items for Systematic Reviews and Meta‐Analyses (PRISMA) guidelines (Page et al., 2021). The PRISMA checklist and an abbreviation table are attached to Tables S1 and S2.

### 2.1 Data sources and searches

Comprehensive searches were conducted in the following electronic databases: PubMed, EMBASE, PsychINFO, and Web of Science from inception through 15 February 2023. The following combinations of keywords with Boolean operators (AND, OR) were applied in the search queries (mental disorders OR depression OR anxiety OR bipolar disorder OR panic disorder OR mood disorders OR affective disorders OR schizophrenia) AND (mean platelet volume OR MPV). We also set limits to the search to identify English‐language trial studies. In addition, we searched the references of all identified articles to obtain additional records. The complete search strategies we applied for identifying relevant articles are available in Table S3.

### 2.2 Inclusion and exclusion criteria

We included studies if they met the following criteria: (1) included patients diagnosed with MDs (depression, BD, anxiety/PD, and SCZ) according to diagnostic criteria or psychiatrists; (2) were original studies using case–control, cross‐sectional, and cohort study designs; (3) recruited healthy controls as a comparison; (4) the mean ± standard deviation (SD) of MPV values or the data for calculation were provided; (5) were published in the English language. We excluded studies that reported duplicate data, that were reviews, comments, or letters, or that included participants with thrombotic or inflammatory disorders that may interfere with MPV values (e.g., coronary artery disease, infection, and severe somatic diseases).

### 2.3 Study selection and data extraction

Two investigators (Z.C. and J.W.) independently reviewed titles, abstracts, and full texts to confirm the eligibility. A third independent investigator (C.C.) discussed and resolved disagreements between the two investigators.

From all identified studies, two independent investigators extracted the first author, year of publication, study design, the sample size, the diagnostic instrument of MDs, region of the survey, the specific diagnoses of MD, APD intake history, sex ratio (male/female), and the mean and SD values of MPV. The third independent investigator (C.C.) verified the data's accuracy.

### 2.4 Quality assessment

Using the Newcastle‐Ottawa Quality Assessment Scale (NOS), we assessed the risk of bias in the included studies (Stang, 2010). Each study's quality was evaluated across three domains: selection (four items), comparability (two items), and exposure/outcome (three items). Each article was assigned a score of 0 or 1, ranging from 1 to 9. Disagreements were resolved through dialog between the two investigators.

### 2.5 Level of evidence

Two investigators (Z.C. and J.W.) independently evaluated each comparison and its outcome using the Grading of Recommendations Assessment Development and Evaluation (GRADE) (Guyatt et al., 2008). The quality of evidence was categorized as high, moderate, low, or very low based on five domains: risk of bias, indirectness, inconsistency, imprecision, and publication bias in the GRADE approaches.

### 2.6 Statistical analysis

We conducted the statistical meta‐analysis using the Stata software (Version 17, Stata Corporation) and GraphPad Prism (Version 9, Dotmatics, the University of California) to draw the figures. Standardized mean difference (SMD) and 95% confidence interval (CI) were applied to analyze the difference in MPV values between patients with MDs and the healthy control group. The heterogeneity across studies was tested by the Cochran *Q* and *I* ^2^ statistics (Higgins, 2003). *I* ^2^ statistics >50% was considered statistically significant heterogeneity. We pooled the SMD of MPV values using a random‐effect model (DerSimonian‐Laird). Conventionally, SMD and effect size (ES) estimates were interpreted as “small” (<0.20), “medium” (0.20–0.80), and “large” (>0.80) (Cohen, 2013). We ran stratified analyses according to specific diagnoses of MD and other factors. Meta‐regression was also performed to detect the potential sources of heterogeneity.

We performed the sensitivity analysis in three ways to verify the certainty and consistency of the results: (a) leave‐one‐out method; (b) excluded studies with small sample size (<100 subjects); (c) excluded studies that recruited patients with APD intake history within 1 month before the sampling of blood. We examined potential bias using Egger's test (Sterne & Egger, 2001). The trim‐and‐fill method (Duval & Tweedie, 2000) was applied to estimate the effect of publication bias on interpreting the outcomes once Egger's test detected a potential publication bias.

## 3 RESULTS

### 3.1 Study selection

**FIGURE 1:**
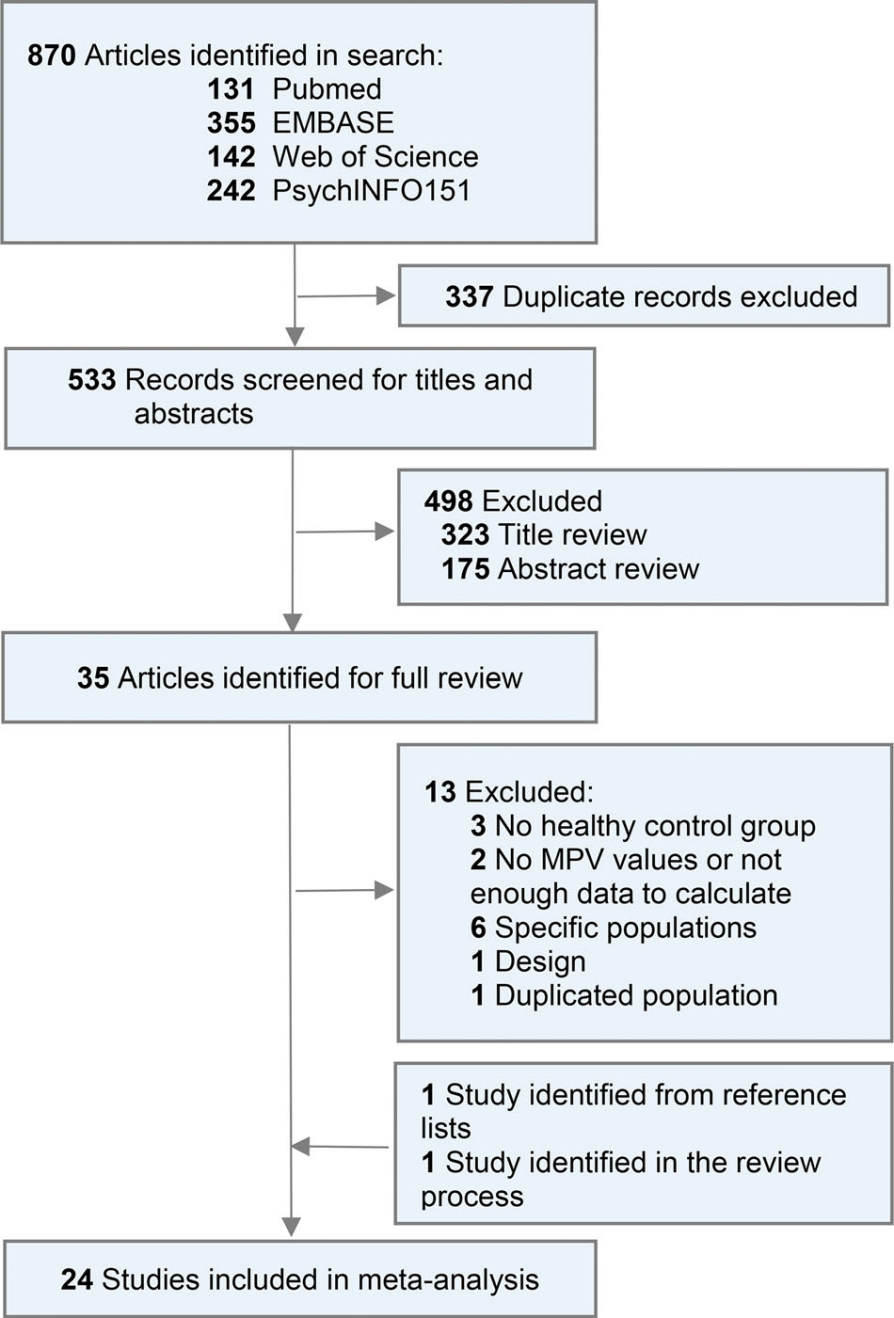
Search flow diagram.

We identified 870 records by our search strategy and removed 337 duplicated records after the screening. Of 533 unique studies, 498 were excluded based on title and abstract review. Thirty‐five articles were relevant for a full‐text review, and 13 were excluded based on the wrong population, absence of healthy controls, duplicated population, and the lack of necessary data. One study was identified from a reference list searching the included studies, whereas another was recognized throughout the review process. Finally, 24 studies met the inclusion criteria and were included in our meta‐analysis (Figure 1). Excluded articles with reasons are shown in Table S4.

### 3.2 Characteristics of included studies

Twenty‐four studies with a total sample of 4843 participants (2450 [50.6%] patients with MDs and 2393 [49.4%] controls) from 5 countries were included. In addition, 23 surveys provided data on the sex ratio of the study population (2048 [43.6%] males and 2651 [56.4%] females), and 23 provided data on the age of the study and control groups (36.8 ± 12.1 vs. 36.2 ± 13.2 years). The diagnoses of common MDs extracted included depression (*n* = 6), anxiety/PD (*n* = 8), BD (*n* = 4), and SCZ (*n* = 6). Most of the studies were conducted in Turkey (*n* = 16), second China (*n* = 3), India (*n* = 3), and lastly Bangladesh (*n* = 1) and Egypt (*n* = 1). Most surveys were case–control designs (*n* = 19), some were cross‐sectional designs (*n* = 4), and one was a prospective cohort study. Table 1 summarizes the descriptive data of the included studies.

**TABLE 1:** Selected characteristics of 24 studies included in meta‐analysis.

			Study		Specific	Subjects		Mean age		MPV	
Source (year)	Country	Year	design	Tools	Diagnosis	Patient	Control	Patient	Control	Patient	Control
Canan et al. (2012)	Turkey		PCS	DSM‐4	MDD	84	575	40.6 ± 13.1	43.8 ± 16.0	9.50 ± 1.80	8.90 ± 1.40
Cai et al. (2017)	China		CCS	DSM‐4	MDD	103	106	46.6 ± 13.4	46.6 ± 12.9	9.39 ± 0.60	9.06 ± 0.85
Ataoglu and Canan (2009)	Turkey		CCS	DSM‐4	MDD	15	17	37.1 ± 10.3	35.6 ± 6.9	10.92 ± 0.75	10.01 ± 0.75
Wang et al. (2022)	China		CCS	ICD‐10	DD	61	30	42.0 ± 10.8	37.1 ± 12.9	9.96 ± 0.93	9.83 ± 0.93
Öztürk et al. (2019)	Turkey		CCS	DSM‐4	MDD	49	48	40.9 ± 14.7	36.9 ± 10.1	9.13 ± 2.13	7.19 ± 1.84
Gündüz et al. (2017)	Turkey		CCS	DSM‐4	MDD	75	57	31.7 ± 10.9	32.4 ± 11.1	8.12 ± 1.70	7.26 ± 1.28
Almis and Aksoy (2018)	Turkey		CCS	DSM‐5	GAD	60	60	39.8 ± 19.0	34.9 ± 14.6	8.15 ± 1.41	7.50 ± 1.24
Bondade et al. (2018)	India		CCS	DSM‐5	AD	76	49	35.1 ± 9.2	34.7 ± 6.8	9.84 ± 1.32	8.77 ± 0.44
Mukta et al. (2021)	Bangladesh		CSS	DSM‐5	GAD	72	72	NA	NA	10.68 ± 0.68	7.51 ± 0.40
Kokacya et al. (2015)	Turkey		CCS	DSM‐4	PD	61	63	23.5 ± 5.4	26.6 ± 4.2	10.12 ± 0.32	8.27 ± 0.91
Göğçegöz Gül et al. (2014)	Turkey		CCS	DSM‐4	PD	37	45	34.1 ± 9.5	35.8 ± 10.0	8.80 ± 0.90	9.20 ± 0.80
Asoglu, Aslan, Imre, et al. (2016)	Turkey		CCS	DSM‐5	PD	30	25	37.0 ± 10.0	36.0 ± 13.0	8.19 ± 1.13	6.85 ± 0.67
Ransing et al. (2017)	India		CCS	DSM‐4	PD	123	133	31.9 ± 11.5	31.3 ± 10.0	7.53 ± 0.93	8.91 ± 1.24
Yalamanchili et al. (2018)	India		CCS	ICD‐10	PD	65	65	31.5 ± 4.5	32.5 ± 9.4	10.02 ± 0.37	6.96 ± 0.99
Mert and Terzi (2016)	Turkey	2016	CCS	DSM‐4	BD	132	135	40.2 ± 12.2	40.1 ± 12.1	9.43 ± 1.03	9.11 ± 0.83
Inanli et al. (2019)	Turkey		CCS	DSM‐4	BD	341	114	36.3 ± 12.3	36.0 ± 8.8	10.42 ± 0.86	10.10 ± 0.80
Kirlioglu et al. (2019)	Turkey		CCS	DSM‐5	BD	48	32	37.1 ± 9.9	38.8 ± 6.7	9.18 ± 1.98	8.96 ± 2.02
Kara et al. (2020)	Turkey		CCS	DSM‐4	BD	68	60	34.7 ± 10.6	32.5 ± 7.7	8.57 ± 1.42	7.92 ± 1.41
Semiz et al. (2013)	Turkey		CCS	DSM‐4	SCZ	25	30	41.3 ± 10.6	41.3 ± 10.6	8.95 ± 0.84	8.10 ± 0.90
Asoglu, Aslan, and Canbolat. (2016)	Turkey		CCS	DSM‐5	SCZ	56	30	34.0 ± 10.0	33.0 ± 10.0	7.17 ± 1.17	6.90 ± 0.69
Aydin et al. (2018)	Turkey		CCS	DSM‐4	SCZ	100	37	37.7 ± 9.4	35.0 ± 7.3	10.34 ± 0.93	9.97 ± 0.97
Yu et al. (2020)	China		CSS	DSM‐5	SCZ	106	120	23.7 ± 1.0	22.6 ± 0.81	11.11 ± 0.11	10.70 ± 0.06
Balcioglu and Kirlioglu (2020)	Turkey	2020	CSS	ICD‐10	SCZ	618	445	39.7 ± 10.6	31.2 ± 9.7	9.31 ± 1.88	8.90 ± 1.98
Ali et al. (2022)	Egypt		CSS	DSM‐5	SCZ	45	45	32.4 ± 7.7	33.0 ± 4.2	9.64 ± 0.84	9.03 ± 1.14

Abbreviations: AD, anxiety disorder; BD, bipolar disorder; CCS, case–control study; CSS, cross‐sectional study; DD, depressive disorder; GAD, generalized anxiety disorder; MDD, major Depressive Disorder; MPV, mean platelet volume; NA, not applicable; PCS, prospective cohort study; PD, panic disorder; SCZ, schizophrenia.

### 3.3 Risk of bias

**FIGURE 2:**
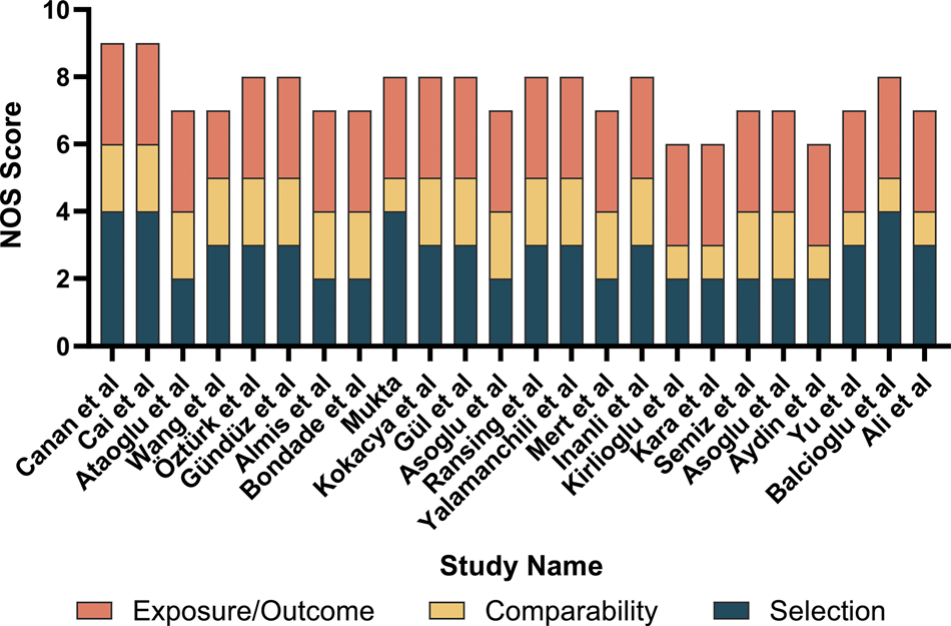
Bar charts of quality assessment.

For 19 case–control studies, 15 (78.9%) did not recruit healthy control from the community. Twelve (63.2%) had potential selection bias in the representativeness of cases. Three (15.8%) had a potential bias in comparability. For four cross‐sectional studies, three (75%) studies did not apply random or continuous sampling. For the prospective cohort study, no potential risks were identified. More details of the quality assessment of each study are shown in Table S5. Twenty‐two (91.7%) scored 7–9, and two (8.3%) were graded 6, respectively (Figure 2).

### 3.4 Overall association between MPV and mental disorders

**FIGURE 3:**
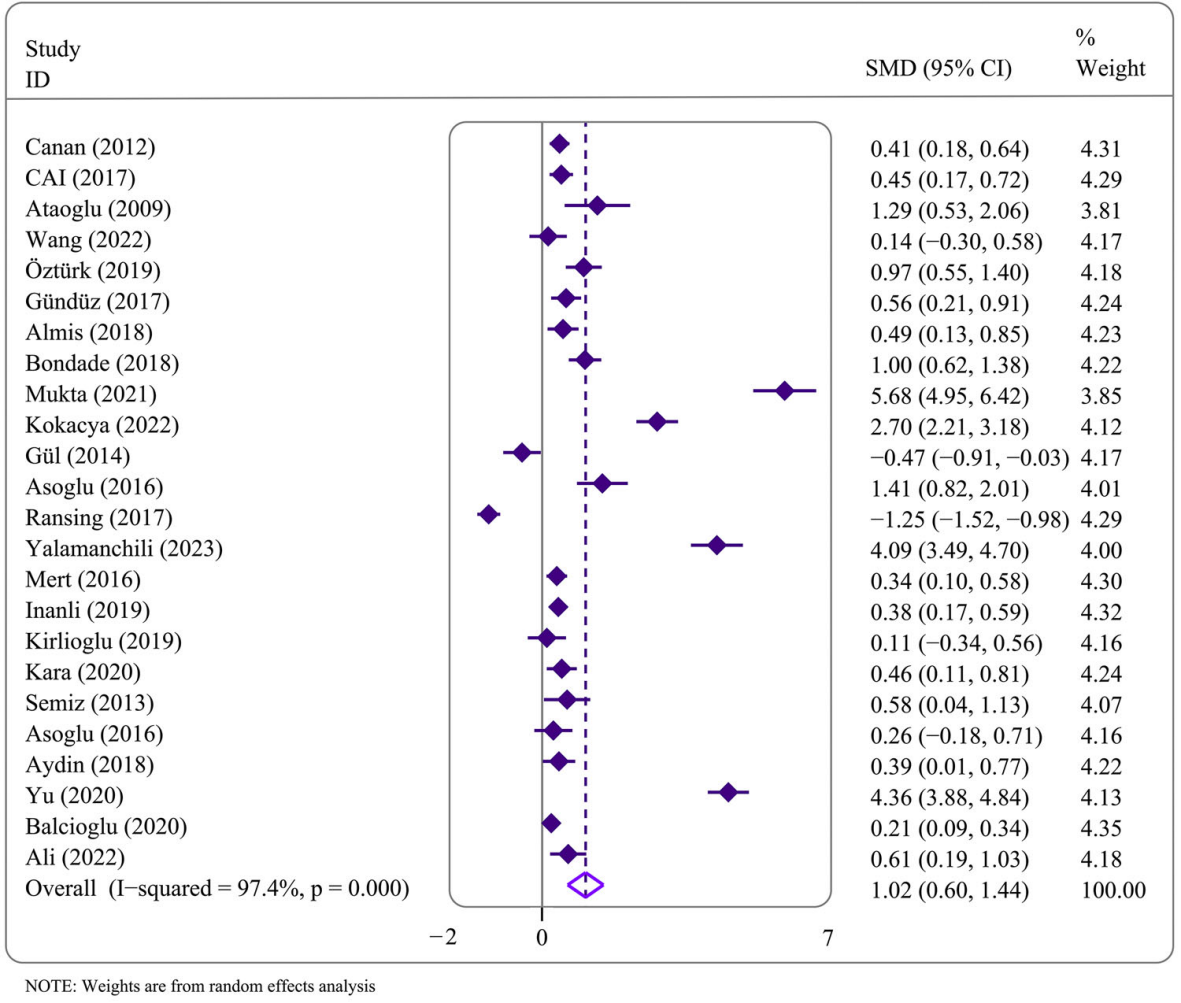
Forest plots of the association between mean platelet volume (MPV) and mental disorders.

**FIGURE 4:**
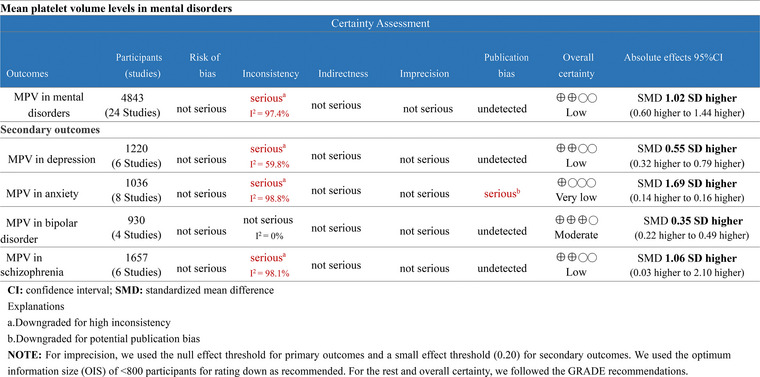
Level of evidence (Grading of Recommendations Assessment Development and Evaluation [GRADE]).

Nineteen of 24 (79.2%) studies reported that patients with MDs had significantly higher MPV values than healthy controls. Conversely, two studies (8.3%) demonstrated higher MPV values in controls. Three studies (12.5%) found no significant difference in MPV levels in the group with MD compared to the control group. The pooled analyses showed a large ES of MPV difference in psychiatric patients compared to controls (SMD = 1.02, 95% CI = .60–1.44, *p* < .001) (Figure 3). However, substantial inter‐survey heterogeneity was found in this outcome (*I* ^2^ = 97.4%, *p* < .001) (Figure 3). GRADE approach reported a low quality of evidence because of substantial heterogeneity across studies (Figure 4).

### 3.5 Subgroup analysis

#### 3.5.1 MPV and depression

**FIGURE 5:**
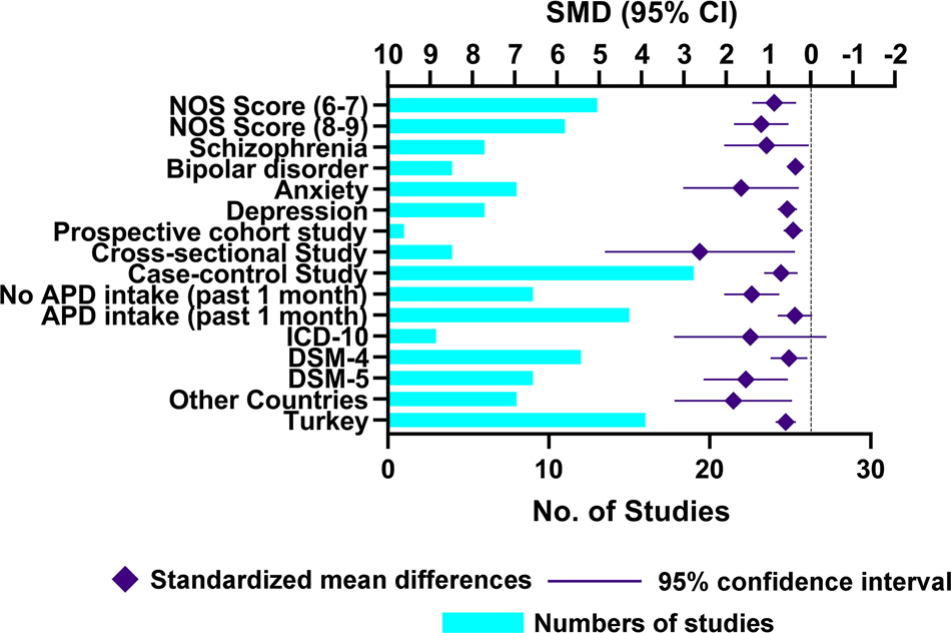
Forest plots and bar charts of stratified analysis. The bar charts represent the number of surveys for review.

Six studies (25%) with 1220 participants with depression reported MPV values. The MPV levels were higher with medium estimates (SMD = .55, 95% CI = .32–.79, *p* < .001, *I* ^2^ = 59.8%) in depressed individuals than in healthy population (Figure 5). No publication bias was detected in the Egger test (*p* = .221). A low quality of evidence was noticed due to the high heterogeneity among studies (Figure 4).

#### 3.5.2 MPV and anxiety/panic disorder

Eight studies (33.3%) provided data for MPV values between subjects with anxiety/PDs and healthy controls, including 524 cases and 512 controls. Pooled analysis showed a large ES of MPV difference in patients with anxiety compared to controls (SMD = 1.69, 95% CI = .27–3.11, *p* = .020, *I* ^2^ = 98.8%) (Figure 5). We detected potential publication bias on the Egger test (*p* = .003) in this outcome. However, the trim‐and‐fill method suggested that this publication bias did not interfere with the estimate of outcomes (no trimming done and data unchanged). Because of potential publication bias and substantial inter‐study heterogeneity, the certainty of evidence was very low quality in this outcome (Figure 4).

#### 3.5.3 MPV and bipolar disorder

Four surveys (16.7%) reported a moderately higher ES of MPV in patients with BD (*n* = 589) than in healthy controls (*n* = 341) (SMD = .35, 95% CI = .22–.49, *p* < .001, *I* ^2^ = 0%) (Figure 5). In addition, no publication bias was detected in the Egger test (*p* = .502). GRADE approach reported a moderate quality of evidence (Figure 4).

#### 3.5.4 MPV and schizophrenia

In 6 surveys (25%), a total of 950 cases with SCZ and 707 controls were enrolled. Based on the pooled ES, MPV had a large effect on the distinction between patients diagnosed with SCZ and controls (SMD = 1.06, 95% CI = .03–2.10, *p* = .044, *I* ^2^ = 98.1%) (Figure 5). The Egger test showed no significant publication bias (*p* = .295). Because of serious limitations in inconsistency, low‐quality evidence was reported by the GRADE approach (Figure 4).

### 3.6 Moderator analysis

The meta‐regression was performed to survey the role of sex (male‐to‐female ratio) and the age of patients with MDs as potential moderators of the association between MPV levels and MDs. The results showed that age was a negative moderator of the association (*β* = −.13, *k* = 23, 95% CI = −.22 to −.04, *p* = .006), suggesting the difference in MVP values between patients with MDs and controls was more dramatic in the younger group. Sex was coded as a continuous variable (male‐to‐female ratio) and did not significantly impact the association between MPV and MD (*β* = −.04, *k* = 23, 95% CI = −.34–.25, *p* = .757) (Table 2).

**TABLE 2:** Summary of meta‐regression and stratified analysis on the association of mean platelet volume (MPV) with mental disorders.

Variables	*N*	SMD(95% CI)	*p* Value	*β*	*I* ^2^(%)	*F*
All	24	1.02 (.60–1.44)	<.001		97.4	
Meta‐regression of mean age on the association of MPV with MDs	23		.006	−.13		
Meta‐regression of males’ proportion on the association of MPV with MDs	23		.757	−.04		
Types			.487			.84
Depression	6	.55 (.32–.79)	<.001		59.8	
Anxiety	8	1.69 (.27–3.11)	.020		98.8	
Bipolar disorder	4	.35 (.22–.49)	< .001		0	
Schizophrenia	6	1.06 (.03–2.10)	.044		98.1	
Regions			.069			3.67
Turkey	16	.59 (.35–.84)	<.001		89.0	
Other countries	8	1.89 (.43–3.31)	.011		99.0	
Tools used to assess mental disorders			.299			1.28
DSM‐4	12	.51 (.06–.96)	.026		95.7	
DSM‐5	9	1.57 (.54–2.61)	.003		97.9	
ICD‐10	3	1.46 (−.41 to 3.33)	.125		98.7	
APD history (1 month before blood test)			.115			2.69
with APD history	9	.39 (−.06 to .78)	.097		94.7	
No APD history	15	1.43 (.75–2.10)	<.001		97.8	
Study design			.065			3.12
Case‐control	19	.71 (.30–1.12)	.001		95.8	
Cross‐sectional	4	2.70 (.37–5.03)	.023		99.3	
Prospective cohort	1	.41 (.18–.64)	.001		NA	
NOS score			.587			.30
Good quality (8–9)	11	1.19 (.53–1.86)	<.001		98.3	
Fair quality (7–6)	13	.87 (.34–1.41)	.001		95.5	

Abbreviations: APD, antipsychotic drug; CI, confidence interval; SMD, standardized mean difference.

We conducted subgroup analyses by the following factors: types, regions, diagnostic instruments, APD intake history, study design, and NOS scores (6–7, 8–9 points). The pooled ESs of MPV did not statistically vary between different MDs (*F* = .84, *p* = .487). In addition, the pooled SMD was not significantly different in studies conducted in Turkey and other countries (China, India, Egypt, and Bangladesh) (SMD: .59 vs. 1.89, *F* = 3.67, *p* = .069). The ESs were similar among groups when stratifying by the type of diagnostic instruments to assess MDs and NOS scores. Finally, for the comparison classified into two subgroups according to APD intake history within 1 month, patients without APD intake history showed significantly higher MPV levels than controls (SMD = 1.43, 95% CI = .75–2.10, *p* < .001), whereas patients with an APD intake history did not (SMD = .39, 95% CI = −.06 to .78, *p* = .097). Table 2 summarizes the pooled ESs for meta‐regression and subgroup analyses.

### 3.7 Sensitivity analysis

We conducted a sensitivity analysis to evaluate the association between MPV and MDs. After the removal of surveys that with a small sample size (<100 subjects), which enrolled patients with APD intake history within 1 month, the outcome remained robust and credible. Furthermore, we used a leave‐one‐out method to estimate the impact of individual studies on the pooled effect. The pooled SMD of MPV varied from .83 (95% CI .45–1.21) to 1.11 (95% CI .72–1.51), suggesting the results of this meta‐analysis were stable and consistent (Table S6).

## 4 DISCUSSION

### 4.1 Main findings

The relationship between inflammatory responses and MDs has become a topic of increasing interest to broader researchers.

As far as we are aware, this is the first quantitative analysis and summary of the association between MPV and MDs. Our studies identified 24 surveys in 5 countries with a combined population of 4843 participants to provide comprehensive estimates of MPV values in patients with common MDs. Included surveys comprised patients with depression, anxiety/PD, BD, and SCZ.

There were several principal findings in our study. We observed significant pooled standard mean differences in MPV values between MDs and non‐MDs individuals. These differences were most evident in patients with anxiety/PD compared to healthy controls. There were no significant differences in MPV found between patients under antipsychotic treatment and controls, suggesting that MPV value can be used as a maker in investigating the effect of antipsychotic treatment. Age was a distinct factor influencing the differences in MPV in moderator analysis. The differences in MPV levels between elderly MD patients and non‐MD controls were reduced than in younger patients.

### 4.2 Platelet and inflammation

There is growing recognition that platelets play a critical role in coordinating immune responses and inflammation. Platelets act rapidly to immune response, and activated platelets release a substantial number of inflammatory mediators that are not involved in hemostasis (Jenne & Kubes, 2015). Platelets contain three primary storage granules: lysosomes, dense granules, and α‐granules, with the latter being the most abundant. Platelet lysosomes possess glycosidases, proteases, and other proteins with a bactericidal effect (Sharda & Flaumenhaft, 2018). Recent research has demonstrated that platelet serotonin plays a vital role in neutrophil aggregation and adhesion to the vascular endothelium (Mauler et al., 2016). Platelet α‐granules possess a variety of proteins that will be released during platelet activation and affect thrombosis, inflammation, and host defenses, among other impacts (Manne et al., 2017).

It is essential to differentiate between acute and chronic inflammation when concentrating on inflammation because platelets appear to switch their effect in the acute setting from pro‐inflammatory to pro‐resorptive. In chronic inflammation, continuously activated platelets release chemokine heteromers (such as platelet factor 4 and heteromers of CC‐chemokine ligand 5) that promote neutrophil adhesion and the release of the intravascular neutrophil extracellular traps. In addition, platelets also engage P‐selectin and interact with P‐selectin glycoprotein ligand 1 (PSGL1), which facilitates neutrophil intravascular migration (Rossaint et al., 2014). Together, platelet–neutrophil interactions provide a complex network and maintain a lower grade but a persistent inflammatory response. Emerging studies reported that in patients with chronic inflammatory diseases (e.g., chronic asthma, chronic urticaria, and psoriasis), researchers observed a more robust platelet activation response, increased platelet aggregation, and increased MPV and distribution width.

### 4.3 Inflammation and mental disorders

Because of the blood–brain barrier, the brain has consistently been recognized as an immune‐released organ. Indeed, massive microglia and astrocytes have been found in the cerebral cortex (Joshi et al., 2019). Nonactivated microglia, known as “quiescent” or “resting” microglia, continuously monitor the environment in non‐pathological circumstances. In response to damage‐associated molecular patterns, such as the P2X7 purinergic receptor (P2X7R), heat shock protein, and histones, activated microglia can be divided into two distinct states: a pro‐inflammatory phenotype M1 and the anti‐inflammatory phenotype M2. Microglia in a pro‐inflammatory phenotype secrete inflammatory signals (such as IL‐1 and TNF‐α), which trigger astrocytes into an activated state (Orihuela et al., 2016). The phenotypically transform‐activated microglia and astrocytes construct a cascaded immune network of amplification and an inflammatory response through their interaction in the brain (Greenhalgh et al., 2020). Recent studies have found that alterations in microglia number and morphology are associated with cognitive and behavioral changes in psychiatric patients (Najjar & Pearlman, 2015; Nakagawa & Chiba, 2014). An increase in macrophage recruitment and microglial activation was observed in the postmortem dorsal anterior cingulate gyrus of the cerebral in individuals suffering from MDD and SCZ (Laskaris et al., 2016; Yirmiya et al., 2015).

### 4.4 Elevated MPV, cardiovascular risk, and mental disorders

It is well documented that patients with depression, anxiety, or disruptive behavior disorder have elevated sympathetic activity, catecholamine levels, and cortisol secretion (Fiedorowicz, 2014; Montaquila et al., 2015; Whooley & Wong, 2013). Meanwhile, catecholamines and sympathetic activity are known to enhance platelet activation (Yun et al., 2016). Platelet activation implies increased volume with higher metabolic and enzymatic activity, containing more prothrombotic substances, and processing increased the expression of thromboxane A2 and B2 and glycoprotein IIb–IIa receptors (Khaspekova 2014). Activated platelets contain more α‐granules that release prothrombotic substances, including platelet and platelet‐derived growth factors, contributing to endovascular hyperplasia (Projahn et al., 2012). Comprehensive meta‐analyses indicated that people with severe mental illness (i.e., BD, SCZ, and MDD) had a significantly increased risk of coronary heart disease versus controls (approximately 50% relative risk higher) (Correll et al., 2017). Our study showed that patients with depression, BD, anxiety and schizophrenia had elevated MPV values than controls. In addition, all included surveys excluded recruiting patients with a history of hypertension, diabetes mellitus, and cardiovascular disease, suggesting elevated MPVs are not associated with chronic somatic disease.

### 4.5 Limitations

Our results have limitations. We did not include non‐English publications or ongoing surveys in our study. High heterogeneity across studies could not be fully explained by moderator analysis. The mean MPV values in both groups differed significantly across studies, which may be related to the difference in the time of blood specimen delivery and the measuring instruments. Most trials were case–control and cross‐sectional studies, and MPV levels during patient follow‐up were unavailable. As most studies in our analysis did not provide data between MPV values and the severity of MDs and treatment status (such as remission and relapse), we cannot draw any conclusions on the above relationship. Most of the studies were from Turkey, China, and India, and data from other countries were warranted.

### 4.6 Clinical implications

MPV is a part of the complete blood count that is simple, inexpensive, and reproducible, yet rarely noticed. In our study, MPV concentrations in individuals with depression, anxiety, BD, and SCZ were higher than in healthy controls, suggesting the involvement of inflammatory response in the pathogenesis of MDs. However, differences in MPV values between psychiatric patients on medication and healthy controls were insignificant, possibly because psychiatric medication is associated with anti‐inflammatory effects. We suggest that MPV value as an add‐on indicator could be integrated into clinical prediction scales for MDs, such as the self‐rating depression scale, Hamilton Anxiety Scale, and bipolar spectrum diagnostic scale, to improve the predictive accuracy and risk stratification of the scales.

## 5 CONCLUSIONS

The results of our meta‐analysis showed that MPV had increased significantly in patients with MDs, suggesting the involvement of inflammatory response in patients with MDs. The difference in MPV was more pronounced in younger psychiatric patients. Meanwhile, the differences in MPV between psychiatric patients with medication and controls were no longer statistically significant, suggesting that MPV may be one of the markers of improvement in patients’ conditions. Further large trials are needed to confirm whether MPV can provide additional value to the diagnosis of psychosis and the determination of treatment outcomes. Until then, the direct use of MPV in evaluating clinical patients is not recommended.

## AUTHOR CONTRIBUTIONS

**Zhichao Chen**: Conceptualization; data curation; formal analysis; investigation; methodology; resources; software; visualization; writing—original draft; writing—review and editing. **Jing Wang**: Conceptualization; data curation; formal analysis; investigation; methodology; resources; software; validation; visualization; writing—original draft; writing—review and editing. **Ciriaco Carru**: Conceptualization; project administration; validation; visualization; writing—review and editing. **Stefania Sedda**: Data curation; investigation; resources; visualization; writing—review and editing. **Alessandra Matilde Nivoli**: Data curation; investigation; resources; visualization; writing—review and editing. **Zhi Li**: Conceptualization; project administration; validation; writing—review, editing and supervision.

## CONFLICT OF INTEREST STATEMENT

The authors declare no conflicts of interest.

### PEER REVIEW

The peer review history for this article is available at https://publons.com/publon/10.1002/brb3.3240.

## Supporting information

Table S1 PRISMA Checklist

Table S2 Lists of abbreviations

Table S3 Search strategies.

Table S4 Reasons for exclusions.

Table S5 Qualities of studies included in meta‐analysis.

Table S6 Sensitivity analysis

## Data Availability

All data analyzed come from published manuscripts.

## References

[brb33240-bib-0001] Ataoglu, A. , & Canan, F. (2009). Mean platelet volume in patients with major depression. Journal of Clinical Psychopharmacology, 29(4), 368–371. 10.1097/jcp.0b013e3181abdfd7 19593177

[brb33240-bib-0002] Asoglu, M. , Aslan, M. , Imre, O. , Kivrak, Y. , Akil, O. , Savik, E. , Buyukaslan, H. , Fedai, U. , & Altindag, A. (2016). Mean platelet volume and red cell distribution width levels in initial evaluation of panic disorder. Neuropsychiatric Disease and Treatment Volume, 12, 2435–2438. 10.2147/ndt.s111108 PMC5036575 27703361

[brb33240-bib-0003] Asoglu, M. , Aslan, M. , & Canbolat, O. (2016). Red cell distribution width, mean platelet volume, and vitamin B12 levels in patients with schizophrenia: An observational study. International Journal of Clinical and Experimental Medicine, 9(8), 16629–16636.

[brb33240-bib-0004] Almis, B. H. , & Aksoy, I. (2018). Mean platelet volume level in patients with generalized anxiety disorder. Psychiatry and Clinical Psychopharmacology, 28(1), 43–47. 10.1080/24750573.2017.1385210

[brb33240-bib-0005] Aydin, M. , Cetin Ilhan, B. , Elmas, T. S. , Cokunlu, Y. , & Eren, I. (2018). Evaluation of mean platelet volume and platelet count in patients with schizophrenia. Family Practice and Palliative Care, 3(2), 102–107. 10.22391/fppc.401101

[brb33240-bib-0006] Ali, E. , Embaby, A. , & Ibrahim, D. (2022). Mean platelet volume, red cell distribution width, and lymphocyte ratios as surrogate predictors of subclinical inflammation in schizophrenia: A cross‐sectional study. EuroMediterranean Biomedical Journal, 17(39), 181–185. 10.3269/1970-5492.2022.17.39

[brb33240-bib-0007] Bandelow, B. , & Michaelis, S. (2015). Epidemiology of anxiety disorders in the 21st century. Dialogues in Clinical Neuroscience, 17, 327–335. 10.31887/DCNS.2015.17.3/bbandelow 26487813 PMC4610617

[brb33240-bib-0008] Bondade, S. , Supriya , Seema, H. S. , & Shivakumar, B. K. (2018). Mean platelet volume in depression and anxiety disorder‐a hospital based case‐control study. International Neuropsychiatric Disease Journal, 11, 1–8. 10.9734/INDJ/2018/42988

[brb33240-bib-0009] Balcioglu, Y. H. , & Kirlioglu, S. S. (2020). C‐reactive protein/albumin and neutrophil/albumin ratios as novel inflammatory markers in patients with schizophrenia. Psychiatry Investigation, 17(9), 902–910. 10.30773/pi.2020.0185 32894927 PMC7538240

[brb33240-bib-0010] Canan, F. , Dikici, S. , Kutlucan, A. , Celbek, G. , Coskun, H. , Gungor, A. , Aydin, Y. , & Kocaman, G. (2012). Association of mean platelet volume with DSM‐IV major depression in a large community‐based population: The MELEN study. Journal of Psychiatric Research, 46(3), 298–302. 10.1016/j.jpsychires.2011.11.016 22154758

[brb33240-bib-0011] Cohen, J. (2013). Statistical power analysis for the behavioral sciences. Academic Press.

[brb33240-bib-0072] Correll, C. U. , Solmi, M. , Veronese, N. , Bortolato, B. , Rosson, S. , Santonastaso, P. , Thapa‐Chhetri, N. , Fornaro, M. , Gallicchio, D. , Collantoni, E. , Pigato, G. , Favaro, A. , Monaco, F. , Kohler, C. , Vancampfort, D. , Ward, P. B. , Gaughran, F. , Carvalho, A. F. , & Stubbs, B. (2017). Prevalence, incidence and mortality from cardiovascular disease in patients with pooled and specific severe mental illness: a large‐scale meta‐analysis of 3,211,768 patients and 113,383,368 controls. World psychiatry: official journal of the World Psychiatric Association (WPA), 16(2), 163–180. 10.1002/wps.20420 28498599 PMC5428179

[brb33240-bib-0012] Crump, C. , Sundquist, K. , Winkleby, M. A. , & Sundquist, J. (2013). Comorbidities and mortality in bipolar disorder. JAMA Psychiatry, 70(9), 931. 10.1001/jamapsychiatry.2013.1394 23863861

[brb33240-bib-0013] Cai, L. , Xu, L. , Wei, L. , & Chen, W. (2017). Relationship of mean platelet volume to MDD: A retrospective study. Shanghai Arch Psychiatry, 29(1), 21–29. 10.11919/j.issn.1002-0829.216082 28769542 PMC5518251

[brb33240-bib-0014] Duval, S. , & Tweedie, R. (2000). Trim and fill: A simple funnel‐plot‐based method of testing and adjusting for publication bias in meta‐analysis. Biometrics, 56(2), 455–463. 10.1111/j.0006-341x.2000.00455.x 10877304

[brb33240-bib-0015] do Espírito Santo, C. C. , Da Silva Fiorin, F. , Ilha, J. , Duarte, M. M. M. F. , Duarte, T. , & Santos, A. R. S. (2019). Spinal cord injury by clip‐compression induces anxiety and depression‐like behaviours in female rats: The role of the inflammatory response. Brain, Behavior, and Immunity, 78, 91–104. 10.1016/j.bbi.2019.01.012 30659938

[brb33240-bib-0016] Fiedorowicz, J. G. (2014). Depression and cardiovascular disease: An update on how course of illness may influence risk. Current Psychiatry Reports, 16, 492. 10.1007/s11920-014-0492-6 25163592 PMC4153989

[brb33240-bib-0017] Guyatt, G. H. , Oxman, A. D. , Vist, G. E. , Kunz, R. , Falck‐Ytter, Y. , Alonso‐Coello, P. , & Schünemann, H. J. (2008). Grade: An emerging consensus on rating quality of evidence and strength of recommendations. BMJ, 336(7650), 924–926. 10.1136/bmj.39489.470347.ad 18436948 PMC2335261

[brb33240-bib-0018] Göğçegöz Gül, I. , Eryılmaz, G. , Ozten, E. , & Sayar, G. H. (2014). Decreased mean platelet volume in panic disorder. Neuropsychiatric Disease and Treatment, 10, 1665. 10.2147/ndt.s69922 25214790 PMC4159394

[brb33240-bib-0019] GBD 2015 Disease and Injury Incidence and Prevalence Collaborators . (2016). Global, regional, and national incidence, prevalence, and years lived with disability for 310 diseases and injuries, 1990–2015: A systematic analysis for the Global Burden of Disease Study 2015. The Lancet, 388(10053), 1545–1602. 10.1016/S0140-6736(16)31678-6 PMC5055577 27733282

[brb33240-bib-0020] Grande, I. , Berk, M. , Birmaher, B. , & Vieta, E. (2016). Bipolar disorder. The Lancet, 387(10027), 1561–1572. 10.1016/S0140-6736(15)00241-X 26388529

[brb33240-bib-0021] Gilman, S. E. , Sucha, E. , Kingsbury, M. , Horton, N. J. , Murphy, J. M. , & Colman, I. (2017). Depression and mortality in a longitudinal study: 1952–2011. Canadian Medical Association Journal, 189(42), E1304–E1310. 10.1503/cmaj.170125 29061855 PMC5654987

[brb33240-bib-0022] Gündüz, N. , Timur, Ö. , Erzincan, E. , Turgut, C. , Turan, H. , & Akbey, Z. Y. (2017). Evalution of mean platelet volume, neutrophile lymphocyte ratio, platelet lymphocyte ratio, and red cell distribution width in patients with major depressive disorder. Medeniyet Medical Journal, 32(4), 230–237. 10.5222/mmj.2017.230

[brb33240-bib-0023] Greenhalgh, A. D. , David, S. , & Bennett, F. C. (2020). Immune cell regulation of glia during CNS injury and disease. Nature Reviews Neuroscience, 21(3), 139–152. 10.1038/s41583-020-0263-9 32042145

[brb33240-bib-0024] Higgins, J. P. T. (2003). Measuring inconsistency in meta‐analyses. BMJ, 327(7414), 557–560. 10.1136/bmj.327.7414.557 12958120 PMC192859

[brb33240-bib-0025] Inanli, I. , Aydin, M. , Çaliskan, A. M. , & Eren, I. (2019). Neutrophil/lymphocyte ratio, monocyte/lymphocyte ratio, and mean platelet volume as systemic inflammatory markers in different states of bipolar disorder. Nordic Journal of Psychiatry, 73(6), 372–379. 10.1080/08039488.2019.1640789 31304832

[brb33240-bib-0026] Jenne, C. N. , & Kubes, P. (2015). Platelets in inflammation and infection. Platelets, 26(4), 286–292. 10.3109/09537104.2015.1010441 25806786

[brb33240-bib-0027] Joshi, A. U. , Minhas, P. S. , Liddelow, S. A. , Haileselassie, B. , Andreasson, K. I. , Dorn, G. W. , & Mochly‐Rosen, D. (2019). Fragmented mitochondria released from microglia trigger A1 astrocytic response and propagate inflammatory neurodegeneration. Nature neuroscience, 22(10), 1635–1648. 10.1038/s41593-019-0486-0 31551592 PMC6764589

[brb33240-bib-0070] Khaspekova, S. G. , Zyuryaev, I. T. , Yakushkin, V. V. , Sirotkina, O. V. , Zaytseva, N. O. , Ruda, M. Y. , Panteleev, M. A. , & Mazurov, A. V. (2014). Relationships of glycoproteins IIb‐IIIa and Ib content with mean platelet volume and their genetic polymorphisms. Blood coagulation & fibrinolysis: an international journal in haemostasis and thrombosis, 25(2), 128–134. 10.1097/MBC.0b013e328364b025 23941967

[brb33240-bib-0028] Kim, H. K. , Andreazza, A. C. , Elmi, N. , Chen, W. , & Young, L. T. (2016). Nod‐like receptor pyrin containing 3 (NLRP3) in the post‐mortem frontal cortex from patients with bipolar disorder: A potential mediator between mitochondria and immune‐activation. Journal of Psychiatric Research, 72, 43–50. 10.1016/j.jpsychires.2015.10.015 26540403

[brb33240-bib-0029] Kokacya, M. H. , Copoglu, U. S. C. , Kivrak, Y. , Ari, M. , Sahpolat, M. , & Ulutas, K. T. (2015). Increased mean platelet volume in patients with panic disorder. Neuropsychiatric Disease and Treatment, 11, 2629–2633. 10.2147/ndt.s94147 26508858 PMC4610766

[brb33240-bib-0030] Köhler, C. A. , Freitas, T. H. , Maes, M. , De Andrade, N. Q. , Liu, C. S. , Fernandes, B. S. , Stubbs, B. , Solmi, M. , Veronese, N. , Herrmann, N. , Raison, C. L. , Miller, B. J. , Lanctôt, K. L. , & Carvalho, A. F. (2017). Peripheral cytokine and chemokine alterations in depression: A meta‐analysis of 82 studies. Acta Psychiatrica Scandinavica, 135(5), 373–387. 10.1111/acps.12698 28122130

[brb33240-bib-0031] Kirlioglu, S. S. , Balcioglu, Y. H. , Kalelioglu, T. , Erten, E. , & Karamustafalioglu, N. (2019). Comparison of the complete blood count‐derived inflammatory markers in bipolar patients with manic and mixed episodes. Bratislava Medical Journal, 120, 195–199. 10.4149/bll_2019_051 31023037

[brb33240-bib-0032] Kara, A. , Karamustafalioglu, N. , Kalelioglu, T. , Genc, A. , & Emul, M. (2020). Platelet mass index as an indicator of platelet activation in manic episode. Neurology Psychiatry and Brain Research, 37, 1–5. 10.1016/j.npbr.2020.04.004

[brb33240-bib-0033] Laskaris, L. E. , Di Biase, M. A. , Everall, I. , Chana, G. , Christopoulos, A. , Skafidas, E. , Cropley, V. L. , & Pantelis, C. (2016). Microglial activation and progressive brain changes in schizophrenia. British Journal of Pharmacology, 173(4), 666–680. 10.1111/bph.13364 26455353 PMC4742288

[brb33240-bib-0034] Montaquila, J. M. , Trachik, B. J. , & Bedwell, J. S. (2015). Heart rate variability and vagal tone in schizophrenia: A review. Journal of Psychiatric Research, 69, 57–66. 10.1016/j.jpsychires.2015.07.025 26343595

[brb33240-bib-0035] Mauler, M. , Bode, C. , & Duerschmied, D. (2016). Platelet serotonin modulates immune functions. Hamostaseologie, 36(01), 11–16. 10.5482/HAMO-14-11-0073 25693763

[brb33240-bib-0036] Mert, D. G. , & Terzi, H. (2016). Mean platelet volume in bipolar disorder: The search for an ideal biomarker. Neuropsychiatric Disease and Treatment, 12, 2057–2062. 10.2147/ndt.s112374 27578978 PMC4998027

[brb33240-bib-0037] Manne, B. K. , Xiang, S. C. , & Rondina, M. T. (2017). Platelet secretion in inflammatory and infectious diseases. Platelets, 28(2), 155–164. 10.1080/09537104.2016.1240766 27848259 PMC5734920

[brb33240-bib-0038] Mukta, F. Y. , Akhter, Q. S. , Azad, A. B. , Layla, K. N. , Akter, T. , Rahman, K. L. , & Sarker, S. (2021). The assessment of platelet indices levels in patients with generalized anxiety disorder. Journal of Biosciences and Medicines, 9(9), 116–124. 10.4236/jbm.2021.99010

[brb33240-bib-0039] Nakagawa, Y. , & Chiba, K. (2014). Role of microglial m1/m2 polarization in relapse and remission of psychiatric disorders and diseases. Pharmaceuticals, 7(12), 1028–1048. 10.3390/ph7121028 25429645 PMC4276905

[brb33240-bib-0040] Najjar, S. , & Pearlman, D. M. (2015). Neuroinflammation and white matter pathology in schizophrenia: Systematic review. Schizophrenia research, 161(1), 102–112. 10.1016/j.schres.2014.04.041 24948485

[brb33240-bib-0041] Olfson, M. , Gerhard, T. , Huang, C. , Crystal, S. , & Stroup, T. S. (2015). Premature mortality among adults with schizophrenia in the United States. JAMA Psychiatry, 72(12), 1172. 10.1001/jamapsychiatry.2015.1737 26509694

[brb33240-bib-0042] Orihuela, R. , Mcpherson, C. A. , & Harry, G. J. (2016). Microglial M1/M2 polarization and metabolic states. British Journal of Pharmacology, 173(4), 649–665. 10.1111/bph.13139 25800044 PMC4742299

[brb33240-bib-0043] Öztürk, A. , Şahan, E. , Mirçik, A. B. , Deveci, E. , Yilmaz, O. , & Kirpinar, I. (2019). Mean platelet volume and neutrophil to lymphocyte ratio decrease in patients with depression with antidepressant treatment. Archives of Clinical Psychiatry (São Paulo), 46, 9–13. 10.1590/0101-60830000000184

[brb33240-bib-0044] Pratt, L. A. , Druss, B. G. , Manderscheid, R. W. , & Walker, E. R. (2016). Excess mortality due to depression and anxiety in the United States: Results from a nationally representative survey. General Hospital Psychiatry, 39, 39–45. 10.1016/j.genhosppsych.2015.12.003 26791259 PMC5113020

[brb33240-bib-0045] Page, M. J. , Mckenzie, J. E. , Bossuyt, P. M. , Boutron, I. , Hoffmann, T. C. , Mulrow, C. D. , Shamseer, L. , Tetzlaff, J. M. , Akl, E. A. , Brennan, S. E. , Chou, R. , Glanville, J. , Grimshaw, J. M. , Hróbjartsson, A. , Lalu, M. M. , Li, T. , Loder, E. W. , Mayo‐Wilson, E. , Mcdonald, S. , … Moher, D. (2021). The PRISMA 2020 statement: An updated guideline for reporting systematic reviews. International Journal of Surgery, 88, 105906. 10.1016/j.ijsu.2021.105906 33789826

[brb33240-bib-0071] Projahn, D. , & Koenen, R. R. (2012). Platelets: key players in vascular inflammation. Journal of leukocyte biology, 92(6), 1167–1175. 10.1189/jlb.0312151 22923486

[brb33240-bib-0046] Rossaint, J. , Herter, J. M. , Van Aken, H. , Napirei, M. , Döring, Y. , Weber, C. , Soehnlein, O. , & Zarbock, A. (2014). Synchronized integrin engagement and chemokine activation is crucial in neutrophil extracellular trap‐mediated sterile inflammation. Blood, 123(16), 2573–2584. 10.1182/blood-2013-07-516484 24335230

[brb33240-bib-0047] Ransing, R. S. , Patil, B. , & Grigo, O. (2017). Mean platelet volume and platelet distribution width level in patients with panic disorder. Journal of Neurosciences in Rural Practice, 08(2), 174–178. 10.4103/jnrp.jnrp_445_16 PMC5402480 28479788

[brb33240-bib-0048] Sterne, J. A. C. , & Egger, M. (2001). Funnel plots for detecting bias in meta‐analysis. Journal of Clinical Epidemiology, 54(10), 1046–1055. 10.1016/S0895-4356(01)00377-8 11576817

[brb33240-bib-0049] Stang, A. (2010). Critical evaluation of the Newcastle‐Ottawa scale for the assessment of the quality of nonrandomized studies in meta‐analyses. European Journal of Epidemiology, 25, 603–605. 10.1007/s10654-010-9491-z 20652370

[brb33240-bib-0050] Semiz, M. , Yücel, H. , & Kavakçı, Ö. , Yildirim, O. , Zorlu, A. , Yilmaz, M. B. , Küçükdurmaz, Z. , & Canan, F. (2013). Atypical antipsychotic use is an independent predictor for the increased mean platelet volume in patients with schizophrenia: A preliminary study. Journal of Research in Medical Sciences: The Official Journal of Isfahan University of Medical Sciences, 18(7), 561–566. 24516487 PMC3897022

[brb33240-bib-0051] Sansanayudh, N. , Anothaisintawee, T. , Muntham, D. , Mcevoy, M. , Attia, J. , & Ammarinthakkinstian, (2014). Mean platelet volume and coronary artery disease: A systematic review and meta‐analysis. International Journal of Cardiology, 175(3), 433–440. 10.1016/j.ijcard.2014.06.028 25017904

[brb33240-bib-0052] Steel, Z. , Marnane, C. , Iranpour, C. , Chey, T. , Jackson, J. W. , Patel, V. , & Silove, D. (2014). The global prevalence of common mental disorders: A systematic review and meta‐analysis 1980–2013. International Journal of Epidemiology, 43(2), 476–493. 10.1093/ije/dyu038 24648481 PMC3997379

[brb33240-bib-0053] Simeone, J. C. , Ward, A. J. , Rotella, P. , Collins, J. , & Windisch, R. (2015). An evaluation of variation in published estimates of schizophrenia prevalence from 1990–2013: A systematic literature review. BMC Psychiatry, 15, 193. 10.1186/s12888-015-0578-7 26263900 PMC4533792

[brb33240-bib-0054] Sharda, A. , & Flaumenhaft, R. (2018). The life cycle of platelet granules. F1000Research, 7, 236. 10.12688/f1000research.13283.1 29560259 PMC5832915

[brb33240-bib-0055] Saghazadeh, A. , Mahmoudi, M. , Shahrokhi, S. , Mojarrad, M. , Dastmardi, M. , Mirbeyk, M. , & Rezaei, N. (2020). Trace elements in schizophrenia: A systematic review and meta‐analysis of 39 studies (N = 5151 participants). Nutrition Reviews, 78(4), 278–303. 10.1093/nutrit/nuz059 31800085

[brb33240-bib-0056] Santa Cruz, E. C. , Madrid, K. C. , Arruda, M. A. Z. , & Sussulini, A. (2020). Association between trace elements in serum from bipolar disorder and schizophrenia patients considering treatment effects. Journal of Trace Elements in Medicine and Biology: Organ of the Society for Minerals and Trace Elements (GMS), 59, 126467. 10.1016/j.jtemb.2020.126467 31954929

[brb33240-bib-0057] Vos, T. , Lim, S. S. , Abbafati, C. , Abbas, K. M. , Abbasi, M. , Abbasifard, M. , Abbasi‐Kangevari, M. , Abbastabar, H. , Abd‐Allah, F. , Abdelalim, A. , Abdollahi, M. , Abdollahpour, I. , Abolhassani, H. , Aboyans, V. , Abrams, E. M. , Abreu, L. G. , Abrigo, M. R. M. , Abu‐Raddad, L. J. , Abushouk, A. I. , … Murray, C. J. L. (2020). Global burden of 369 diseases and injuries in 204 countries and territories, 1990–2019: A systematic analysis for the global burden of disease study 2019. The Lancet, 396(10258), 1204–1222. 10.1016/s0140-6736(20)30925-9 PMC7567026 33069326

[brb33240-bib-0058] Whooley, M. A. , & Wong, J. M. (2013). Depression and cardiovascular disorders. Annual Review of Clinical Psychology, 9, 327–354. 10.1146/annurev-clinpsy-050212-185526 23537487

[brb33240-bib-0059] Walker, E. R. , Mcgee, R. E. , & Druss, B. G. (2015). Mortality in mental disorders and global disease burden implications: a systematic review and meta‐analysis. JAMA Psychiatry, 72(4), 334. 10.1001/jamapsychiatry.2014.2502 25671328 PMC4461039

[brb33240-bib-0060] Wieck, A. , Grassi‐Oliveira, R. , Do Prado, C. H. , Viola, T. W. , Petersen, L. E. , Porto, B. , Teixeira, A. L. , & Bauer, M. E. (2016). Toll‐like receptor expression and function in type I bipolar disorder. Brain, Behavior, and Immunity, 54, 110–121. 10.1016/j.bbi.2016.01.011 26795430

[brb33240-bib-0061] Wang, J.‐M. , Yang, K.‐D. , Wu, S.‐Y. , Zou, X.‐G. , Liao, Y.‐S. , Yang, B. , Xie, B.‐N. , Huang, Y. , Li, S.‐J. , & Ma, H.‐J. (2022). Platelet parameters, C‐reactive protein, and depression: An association study. International Journal of General Medicine, 15, 243–251. 10.2147/ijgm.s338558 35023962 PMC8747525

[brb33240-bib-0062] World Health Organization . (2022). International classification of diseases 11th revision. World Health Organization. https://icd.who.int/en/

[brb33240-bib-0063] Yavuz, S. , & Ece, A. (2014). Mean platelet volume as an indicator of disease activity in juvenile SLE. Clinical Rheumatology, 33, 637–641. 10.1007/s10067-014-2540-3 24567240

[brb33240-bib-0064] Yirmiya, R. , Rimmerman, N. , & Reshef, R. (2015). Depression as a microglial disease. Trends in Neurosciences, 38(10), 637–658. 10.1016/j.tins.2015.08.001 26442697

[brb33240-bib-0065] Yun, S.‐H. , Sim, E.‐H. , Goh, R.‐Y. , Park, J.‐I. , & Han, J.‐Y. (2016). Platelet activation: The mechanisms and potential biomarkers. BioMed Research International, 2016, 9060143. 10.1155/2016/9060143 27403440 PMC4925965

[brb33240-bib-0066] Yu, Q. , Weng, W. , Zhou, H. , Tang, Y. , Ding, S. , Huang, K. , & Liu, Y. (2020). Elevated platelet parameter in first‐episode schizophrenia patients: A cross‐sectional study. Journal of Interferon & Cytokine Research, 40(11), 524–529. 10.1089/jir.2020.0117 33121305

[brb33240-bib-0067] Yalamanchili, S. , Pasupula, S. , & Chilukuri, R. (2018). Clinical profile and changes in values of mean platelet volume among panic disorder patients. Archives of Mental Health, 19(1), 15–18. 10.4103/amh.amh_4_18

[brb33240-bib-0068] Zareifar, S. , Farahmand Far, M. R. , Golfeshan, F. , & Cohan, N. (2014). Changes in platelet count and mean platelet volume during infectious and inflammatory disease and their correlation with ESR and CRP. Journal of Clinical Laboratory Analysis, 28(3), 245–248. 10.1002/jcla.21673 24478177 PMC6807431

