# Supplementary material for: Meta‐analysis of peripheral mean platelet volume in patients with mental disorders: Comparisons in depression, anxiety, bipolar disorder, and schizophrenia
Source: Brain Behav. 2023 Aug 29;13(11):e3240. doi: 10.1002/brb3.3240 (PMC10636414; doi:10.1002/brb3.3240)
Supplement: Supplementary file 2 — Table S2 Lists of abbreviations [file BRB3-13-e3240-s005.docx]

| **TABLE S2** | |
| --- | --- |
| **Medical terms** | **Abbreviations** |
| Mental disorders | MDs |
| Mean platelet volume | MPV |
| Standardized mean difference | SMD |
| Confidence interval | CI |
| Bipolar disorder | BD |
| Schizophrenia | SCZ |
| Major depressive disorder | MDD |
| Tumor necrosis factor | TNF |
| Interleukin 6 | IL-6 |
| Interleukin 10 | IL-10 |
| Interferon-γ | IFN-γ |
| Toll-like receptors | TLRs |
| NLR family pyrin domain containing 3 | NLRP3 |
| Panic disorder | PD |
| antipsychotic drug | APD |
| Preferred Reporting Items for Systematic Reviews and Meta-Analyses | PRISMA |
| Standard deviation | SD |
| Newcastle-Ottawa Quality Assessment Scale | NOS |
| Grading of Recommendations Assessment Development and Evaluation | GRADE |
| Effect size | ES |
| Platelet factor 4 | PF4 |
| CC-chemokine ligand 5 | CCL5 |
| Neutrophil extracellular traps | NETs |
| P-selectin glycoprotein ligand 1 | PSGL1 |
| Damage-associated molecular patterns | DAMPs |
| P2X7purinergic receptor | P2X7R |
| Self-Rating Depression Scale | SDS |
| Hamilton Anxiety Scale | HAMA |
| Bipolar Spectrum Diagnostic Scale | BSDS |
| Prospective cohort study | PCS |
| Case-control study | CCS |
| Cross-sectional study | CSS |
| Depressive disorder | DD |
| Generalized anxiety disorder | GAD |
| Anxiety disorder | AD |
