# Supplementary material for: Meta‐analysis of peripheral mean platelet volume in patients with mental disorders: Comparisons in depression, anxiety, bipolar disorder, and schizophrenia
Source: Brain Behav. 2023 Aug 29;13(11):e3240. doi: 10.1002/brb3.3240 (PMC10636414; doi:10.1002/brb3.3240)
Supplement: Supplementary file 3 — Table S3 Search strategies. [file BRB3-13-e3240-s006.docx]

| **TABLE S3** Search strategies | | | |
| --- | --- | --- | --- |
| **Database** | **Search** | **Filters/Limits** | **Numbers**  **of Results** |
| PubMed | (Mental disorders/mean platelet volume | English | 92 |
|  | ((((Depression/mean platelet volume) OR (Anxiety/mean platelet volume)) OR (Bipolar disorder/mean platelet volume)) OR (Panic disorder/mean platelet volume)) OR (Schizophrenia/mean platelet volume) | English | 22 |
|  | (mood disorders/mean platelet volume) OR (affective disorders/mean platelet volume) | English | 17 |
| EMBASE | ('mental disorders'/exp OR 'mental disorders' OR (mental AND ('disorders'/exp OR disorders))) AND 'mean platelet volume':ti,ab,kw | English | 186 |
|  | (('depression'/exp OR depression) AND 'mean platelet volume':ti,ab,kw) OR (('anxiety disorder'/exp OR 'anxiety disorder') AND 'mean platelet volume':ti,ab,kw) OR (('bipolar disorder'/exp OR 'bipolar disorder') AND 'mean platelet volume':ti,ab,kw) OR ('panic disorder'/exp OR 'panic disorder' OR (('panic'/exp OR panic) AND ('disorder'/exp OR disorder))) AND 'mean platelet volume':ti,ab,kw) OR (('schizophrenia'/exp OR schizophrenia) AND 'mean platelet volume':ti,ab,kw) | English | 117 |
|  | (('mood disorder'/exp OR 'mood disorder') AND 'mean platelet volume':ti,ab,kw) OR (('affective disorder'/exp OR 'affective disorder' OR (affective AND ('disorder'/exp OR disorder))) AND 'mean platelet volume':ti,ab,kw) | English | 52 |
| Web of Science | (TS=(Mental disorders) OR TS=(mood disorders) OR TS= ('affective disorders)) AND ((TS=(mean platelet volume)) OR TS=(MPV)) | English | 36 |
|  | (((((TS=(depression)) OR TS=(anxiety disorder)) OR TS=(bipolar disorder)) OR TS=(panic disorder)) OR TS=(schizophrenia)) AND (  (TS=(mean platelet volume)) OR TS=(MPV)) | English | 106 |
| PsychINFO | TX (mental disorders ) AND TX ( (mean platelet volume) OR (MPV) ) | English | 77 |
|  | TX ( mood disorders OR affective disorders ) AND TX ( (mean platelet volume) OR (MPV) ) | English | 75 |
|  | TX ( depression OR anxiety OR panic disorder OR bipolar disorder OR schizophrenia ) AND TX ( (mean platelet volume) OR (MPV) ) | English | 90 |
