# Supplementary material for: Meta‐analysis of peripheral mean platelet volume in patients with mental disorders: Comparisons in depression, anxiety, bipolar disorder, and schizophrenia
Source: Brain Behav. 2023 Aug 29;13(11):e3240. doi: 10.1002/brb3.3240 (PMC10636414; doi:10.1002/brb3.3240)
Supplement: Supplementary file 4 — Table S4 Reasons for exclusions. [file BRB3-13-e3240-s002.docx]

| **TABLE S4** Reasons for exclusions | | | |
| --- | --- | --- | --- |
| No. | First Author, year | Reasons | Details |
| 1. | Williams MS_2014^1^ | Specific population | Enrolled patients with acute coronary syndromes |
| 2. | Fusar-Poli L_2021^2^ | Measurement | No MPV values or not enough data to calculate |
| 3. | Wysokiński A_2016^3^ | Control | No healthy control group |
| 4. | Gialluisi A_2020^4^ | Design | Letter to the editor |
| 5. | Young JG_1980^5^ | Measurement | No MPV values or not enough data to calculate |
| 6. | Abu-Zeid AM_2020^6^ | Specific population | Participants with chronic kidney disease |
| 7. | Jain N_2019^7^ | Specific population | Investigated in patients with chronic kidney disease |
| 8. | Fábián B_2020^8^ | Specific population | Enrolled patients with autoimmune disorders |
| 9. | Lee J_2014^9^ | Control | No healthy control group |
| 10. | Qiu H_2018^10^ | Specific population | Patients with acute ischemic stroke |
| 11. | Atagün Mİ_2016^11^ | Control | No healthy control group |
| 12. | Wibawa A_2015^12^ | Specific population | Sampling was carried out from Stable Angina Pectoris patients |
| 13. | Inanli I_2021^13^ | Population | Conducted on duplicated population |

1. Williams MS, Rogers HL, Wang NY, et al. Do platelet-derived microparticles play a role in depression, inflammation, and acute coronary syndrome [J]. Psychosomatics, 2014, 55(3): 252-260.
2. Fusar-Poli L, Natale A, Amerio A, et al. Neutrophil-to-lymphocyte, platelet-to-lymphocyte and monocyte-to-lymphocyte ratio in bipolar disorder[J]. Brain Sciences, 2021, 11(1): 58.
3. Wysokiński A, Szczepocka E. Platelet parameters (PLT, MPV, P-LCR) in patients with schizophrenia, unipolar depression and bipolar disorder[J]. Psychiatry research, 2016, 237: 238-245.
4. Gialluisi A, Izzi B, Di Castelnuovo A, et al. Revisiting the link between platelets and depression through genetic epidemiology: new insights from platelet distribution width[J]. Haematologica, 2020, 105(5): e246.
5. Young JG, Cohen DJ, Waldo MC, et al. Platelet monoamine oxidase activity in children and adolescents with psychiatric disorders[J]. Schizophrenia Bulletin, 1980, 6(2): 324-333.
6. Abu-Zeid AM, Morsy SM, Mohamed SH. Study of the Mean Platelet Volume in Children with Chronic Renal Diseases[J]. The Egyptian Journal of Hospital Medicine, 2020, 81(2): 1342-1346.
7. Jain N, Wan F, Kothari M, et al. Association of platelet function with depression and its treatment with sertraline in patients with chronic kidney disease: Analysis of a randomized trial[J]. BMC nephrology, 2019, 20(1): 1-13.
8. Fábián B, Horváth I F, Shemirani A H, et al. Depression and Anxiety Symptoms Are Associated with Mean Platelet Volume in Autoimmune Disorders[J]. International journal of environmental research and public health, 2022, 19(17): 11006.
9. Lee J, Powell V, Remington G. Mean platelet volume in schizophrenia unaltered after 1 year of clozapine exposure[J]. Schizophrenia research, 2014, 157(1-3): 134-136.
10. Qiu H, Liu Y, He H, et al. The association between mean platelet volume levels and poststroke depression[J]. Brain and Behavior, 2018, 8(10): e01114.
11. Atagün Mİ, Korkmaz ŞA, SoykanÇ, et al. Serum lithium levels are associated with white blood cell counts in bipolar disorder[J]. Turkish Journal of Medical Sciences, 2016, 46(4): 1271-1272.
12. Wibawa A, Siswanto A, Hariawan H. The Difference of Average Mean Platelet Volume (MPV) Values in Stable Angina Pectoris Patients with Symptoms of Depression and without Symptoms of Depression[J]. Acta Interna: The Journal of Internal Medicine, 5(1): 28-34.
13. Inanli I, Caliskan A M, Aydin M, et al. Evaluation of inflammatory markers in bipolar disorder: A comparative study[J]. Medicine, 2021, 10(4): 1362-7.
