# Supplementary material for: Meta‐analysis of peripheral mean platelet volume in patients with mental disorders: Comparisons in depression, anxiety, bipolar disorder, and schizophrenia
Source: Brain Behav. 2023 Aug 29;13(11):e3240. doi: 10.1002/brb3.3240 (PMC10636414; doi:10.1002/brb3.3240)
Supplement: Supplementary file 5 — Table S5 Qualities of studies included in meta‐analysis. [file BRB3-13-e3240-s001.docx]

| **TABLE S5** Qualities of studies included in meta-analysis. | | | | | | | | | | |
| --- | --- | --- | --- | --- | --- | --- | --- | --- | --- | --- |
| **Study name** | **Selection** | | | | **Comparability** | | **Exposure/Outcome** | | | |
|  | **Q1** | **Q2** | **Q3** | **Q4** | **Q5** | **Q6** | **Q7** | **Q8** | **Q9** | **Score** |
| Canan et al | Y | Y | Y | Y | Y | Y | Y | Y | Y | 9 |
| Cai et al | Y | Y | Y | Y | Y | Y | Y | Y | Y | 9 |
| Ataoglu et al | Y | Y | N | N | Y | Y | Y | Y | Y | 7 |
| Wang et al | Y | Y | N | Y | Y | Y | Y | Y | N | 7 |
| Öztürk et al | Y | Y | N | Y | Y | Y | Y | Y | Y | 8 |
| Gündüz et al | Y | Y | N | Y | Y | Y | Y | Y | Y | 8 |
| Almis et al | Y | N | N | Y | Y | Y | Y | Y | Y | 7 |
| Bondade et al | Y | N | N | Y | Y | Y | Y | Y | Y | 7 |
| Kokacya et al | Y | Y | N | Y | Y | Y | Y | Y | Y | 8 |
| Gül et al | Y | N | Y | Y | Y | Y | Y | Y | Y | 8 |
| Asoglu et al | Y | N | N | Y | Y | Y | Y | Y | Y | 7 |
| Ransing et al | Y | N | Y | Y | Y | Y | Y | Y | Y | 8 |
| Yalamanchili et al | Y | Y | N | Y | Y | Y | Y | Y | Y | 8 |
| Mert et al | Y | N | N | Y | Y | Y | Y | Y | Y | 7 |
| Inanli et al | Y | N | Y | Y | Y | Y | Y | Y | Y | 8 |
| Kirlioglu et al | Y | N | N | Y | N | Y | Y | Y | Y | 6 |
| Kara et al | Y | N | N | Y | N | Y | Y | Y | Y | 6 |
| Semiz et al | Y | N | N | Y | Y | Y | Y | Y | Y | 7 |
| Aydin et al | Y | N | N | Y | Y | N | Y | Y | Y | 6 |
| Asoglu et al | Y | N | N | Y | Y | Y | Y | Y | Y | 7 |
|  | **Selection** | | | | **Comparability** | | **Outcome** | | |  |
|  | **Q1** | **Q2** | **Q3** | **Q4** | **Q5** |  | **Q6** |  | **Q7** |  |
| Mukta et al | Y | N | Y | YY | Y |  | YY |  | Y | 8 |
| Yu et al | N | N | Y | YY | Y |  | YY |  | Y | 7 |
| Balcioglu et al | N | Y | Y | YY | Y |  | YY |  | Y | 8 |
| Ali et al | N | N | Y | YY | Y |  | YY |  | Y | 7 |
| Y, Yes; N, No or Not specified. | | | | | | | | | | |
