# Supplementary figures and images for: Meta‐analysis of peripheral mean platelet volume in patients with mental disorders: Comparisons in depression, anxiety, bipolar disorder, and schizophrenia
Source: Brain Behav. 2023 Aug 29;13(11):e3240. doi: 10.1002/brb3.3240 (PMC10636414; doi:10.1002/brb3.3240)

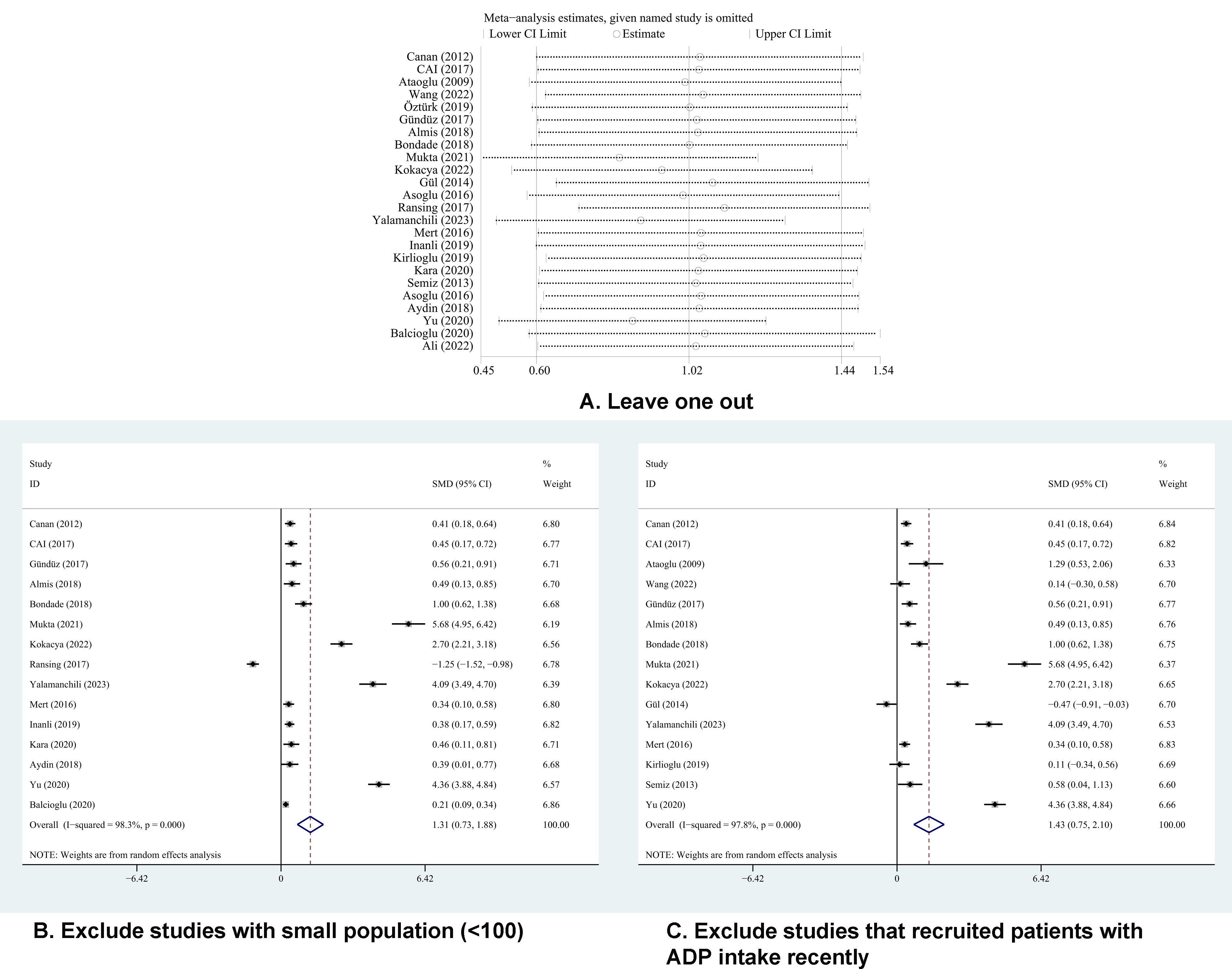

Supplement: Supplementary file 6 — Table S6 Sensitivity analysis [file BRB3-13-e3240-s003.docx]
